# National trends in incidence and survival of chronic lymphocytic leukemia in Norway for 1953–2012: a systematic analysis of population‐based data

**DOI:** 10.1002/cam4.849

**Published:** 2016-11-04

**Authors:** Andrea Lenartova, Tom Børge Johannesen, Geir Erland Tjønnfjord

**Affiliations:** ^1^ Department of HaematologyOslo University HospitalP.O.Box 4950 NydalenOslo0424Norway; ^2^ The Institute of Clinical MedicineFaculty of MedicineUniversity of OsloOsloNorway; ^3^ The Cancer Registry of NorwayP.O. Box 5313 MajorstuenOslo0304Norway

**Keywords:** chronic lymphocytic leukemia, cancer registry, national incidence, net cancer survival

## Abstract

Chronic lymphocytic leukemia is a disease of the elderly, and despite major advances in treatment, remains incurable. The Cancer Registry of Norway has registered data on patients with chronic lymphocytic leukemia since 1953. We aimed to analyze trends in incidence and survival of chronic lymphocytic leukemia in Norway. We identified 7664 patients reported with chronic lymphocytic leukemia to the registry between 1953 and 2012. We gathered information on sex, age at diagnosis, date of death and basis for diagnosis. The age‐standardized incidence increased from 0.6/100.000 person‐years in 1953 to 3.1/100,000 person‐years in 2012. We found a significant decrease in median age between 1993–2002 and 2003–2012 (75 vs. 72 years, 95%CI: 2.52–3.98, *P* < 0.001). Men were diagnosed at a significantly younger age than women. Immunophenotyping has become the most important diagnostic method after 2002. Median observed survival increased from 3 years in 1952–1963 to 8.5 years in 2003–2012. Five‐ and 10‐year age‐standardized net survival increased throughout the whole period across age groups and reached 79% and 57%, respectively. Median observed survival was significantly shorter in men than in women in 1993–2002 (4.9 vs. 6.1 years, *P* < 0.001). The gap between survival rates for men and women was diminishing in 2003–2012 in patients younger than 60 years while it remained considerable in older patients. Despite an aging Norwegian population, chronic lymphocytic leukemia (CLL) patients become younger at diagnosis. A fourfold increase in incidence, a prolonged survival, and major changes in diagnostic methods in Norway were observed.

## Introduction

Major advances in diagnosis and treatment of chronic lymphocytic leukemia (CLL) emerged during the last decades. Definitive diagnosis is easily established by flow cytometry of peripheral blood with no need for histological confirmation. The International Workshop on Chronic Lymphocytic Leukemia 2008 guidelines for the diagnosis of CLL requires a monoclonal B‐lymphocyte count of 5 x 10^9^/L or more and a characteristic cell‐surface phenotype of B cells: the presence of CD5, CD19, and CD23, weak expression of CD20 and CD79b, and either kappa or lambda immunoglobulin light chains. The definition of small lymphocytic lymphoma (SLL) requires the presence of lymphadenopathy and/or splenomegaly and clonal B lymphocytes in the peripheral blood should not exceed 5 × 10^9^/L 1.

CLL is a disease of the elderly, and the population is aging in the Western world. Incidence of CLL is highest among Caucasians, intermediate among Africans/African Americans, and low among Asian/Pacific Islanders 2. Annual incidence of 2–6 per 100.000 in the general population has been reported. Epidemiologic studies report divergent trends of incidence, increasing in Denmark and USA, and stable in Sweden 3, 4, 5. The widespread use of immunophenotyping seems to increase the incidence of CLL 6, 7. Standard tumor registries do not monitor flow cytometry reports and may miss incidentally diagnosed asymptomatic patients.

No curative standard treatment for CLL is currently available. Chemoimmunotherapy with fludarabine, cyclophosphamide, and rituximab or alemtuzumab improves progression‐free survival and overall survival in fit patients 8, 9.

Results of trials investigating the efficacy of chlorambucil in combination with anti‐CD20 antibodies show prolonged progression‐free and overall survival 10, 11, 12. Recently, the small molecular inhibitors of kinases lend support to targeted therapy. Precise and easily available diagnostic methods and possible curative, well‐tolerated treatment may make call on population screening.

Accurate incidence and survival data for CLL in Norway have not been published previously. The aim of this retrospective study was to assess CLL incidence and survival patterns in Norway based on information reported to the population‐based nationwide Cancer Registry of Norway between 1953 and 2012.

## Methods

### Central registries

The Cancer Registry of Norway, established in 1952, is considered close to 100% completion 13. Reporting is regulated by Norwegian law and is mandatory for all health care providers. The registry receives copies of all pathology and autopsy reports, registration forms filled in by the clinicians, copies of all death certificates that mention neoplastic disease, hospital discharge data, outpatient diagnosis, and radiotherapy data. The database is matched to information from the Norwegian Cause of Death Registry at Statistics Norway and to the National Registry on Vital Status and Migration.

### Patient cohort

We identified all patients reported with CLL and SLL to the Cancer Registry of Norway between January 1, 1953 and December 31, 2012. The date of death was extracted from the Norwegian Cause of Death Registry.

We excluded 10 children aged 1–12 years after reviewing their pathology reports sent in at registration. The children were diagnosed with acute lymphoblastic leukemia but miscoded and registered as CLL.

We did not exclude patients on the basis of previous cancer diagnoses. Lymphoma classification before 1968 includes the future entity SLL under “Lymphocytic lymphosarcoma” code 14. This entity included several other, now well‐described lymphomas such as mantel cell lymphoma which are not identical with SLL. Only diagnoses identical with the future entity SLL:B‐lymphocyte malignant non‐Hodgkin lymphoma, coded from 1968 are included in the study 15. We reviewed 133 cases that were consecutively reported with code “B‐cell lymphoma not otherwise specified”. All, except two reports stated SLL and confirmed CLL/SLL diagnosis. In two cases, the previous CLL/SLL diagnosis was reconsidered to be rather lymphoplasmacytic lymphoma on the basis of reassessment by the pathologist.

Information on sex, date of birth, date of diagnosis, basis for diagnosis, date of death was gathered from the national registries. The basis for diagnosis was grouped in categories. “Immunophenotyping” includes flow cytometry, immunohistochemistry, and immunofluorescence. “Cytology” includes microscopy of blood and bone marrow smears performed by the clinicians in Norway and microscopy of fine needle aspiration material performed by the pathologists. “Histology” includes microscopy of bone marrow, lymph node, and tumor biopsy performed by the pathologists. “Autopsy” includes autopsy‐based diagnosis. “Other” includes clinical and radiological diagnosis, death certificate‐based diagnosis, and unknown basis for diagnosis. Cytogenetics and molecular genetics registered as source of diagnosis are included in this category.

All patients were observed from date at diagnosis until death or 15.6.2015. Eleven patients emigrated and their data were censored at the time of emigration. Median follow‐up was 12.3 years (95% CI: 12.1–12.6 years) derived by “reversed Kaplan–Meier” method.

The Regional Committee for Medical and Health Research Ethics of South‐East Norway approved the study (Reg. nr. 2014/427/REK sør‐øst).

### Statistical analysis

Student *t*‐test and comparison of two proportions were used to compare demographic data.

Denominator age‐specific person‐years for incidence rates were estimated from the Norwegian population data. Incidence rates were age‐standardized to the world standard population 16. We used Joinpoint Regression Program to test whether apparent change in age‐adjusted incidence trends is statistically significant (Version 4.1.1 ‐ August 2014; Statistical Methodology and Applications Branch, Surveillance Research Program, National Cancer Institute, USA) 17. The tests of significance in this software use the Monte Carlo permutation method.

Absolute survival was estimated by the Kaplan–Meier method and compared by the log‐rank test. We calculated both cumulative net survivals by the Pohar‐Perme method and relative survival rates by the Ederer‐II method 18, 19. The expected mortality rates of the Norwegian population were available by sex, age, and year of diagnosis at Statistics Norway. Age‐standardized net survival estimates were calculated using the international cancer survival standard weights 20.

Five hundred and fifteen patients diagnosed at autopsy or registered by death certificate only were excluded from the survival analysis.

Calculations were performed using STATA SE 14 (StataCorp, College Station, TX).

## Results

### The study population

**Figure 1:**
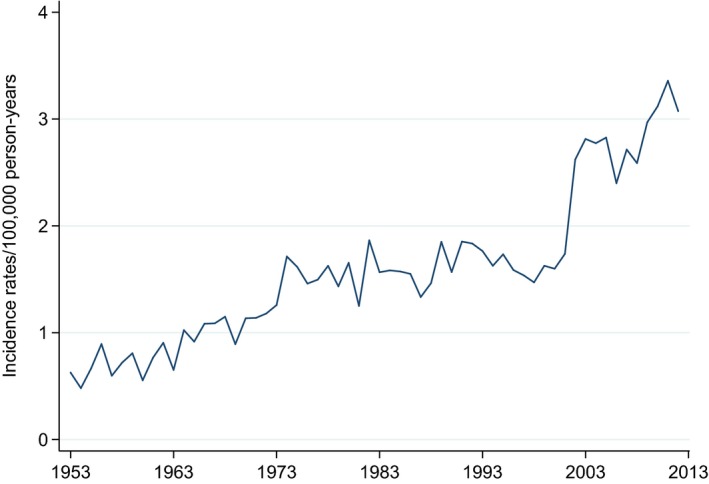
Age‐standardized incidence rates/100.000 person‐years, 1953–2012 (World standard population).

Between January 1, 1953 and December 31, 2012, a total of 7664 patients with CLL and SLL were registered at the Cancer Registry of Norway (Table 1). Median age at diagnosis increased gradually from 68 years in 1953–1962 to 75 years in 1993–2002 (Table S1). Subsequently, there was a significant shift toward a lower median age in 2003–2012 (75 vs. 71.8 year, difference 3.25 year, CI: 2.52–3.98, *P* < 0.001). Proportion of patients younger than 70 years increased significantly in 2003–2012 compared to 1993–2002 (45% vs. 33%; *P* < 0.001). The youngest patient diagnosed with CLL was 25 years old, the eldest 102 years old. Figure 1.

**Table 1:** Number of patients with CLL by age group, sex, and calendar period

Periods	Age group				Men (%)	Women	Total
	0–59 (%)	60–69 (%)	70–79 (%)	80+ (%)			
1953–1962	81 (23)	133 (38)	103 (29)	34 (10)	203 (58)	148	351
1963–1972	130 (20)	215 (34)	207 (32)	90 (14)	402 (63)	240	642
1973–1982	190 (17)	312 (27)	386 (34)	251 (22)	685 (60)	454	1139
1983–1992	174 (13)	347 (26)	471 (35)	347 (26)	755 (56)	584	1339
1993–2002	195 (13)	317 (20)	544 (35)	501 (32)	904 (58)	653	1557
2003–2012	480 (18)	718 (27)	754 (29)	684 (26)	1524 (58)	1112	2636
**1953–2012**	1250 (16)	2042 (27)	2465 (32)	1907 (25)	4473 (58)	3191	7664

Bold indicates 60 year summarized

The proportion of men (56–63%) remained fairly stable throughout the study period (Table 1). Men were diagnosed at a significantly younger age than women after 1963–1972 (71 vs. 74 year, respectively). The age difference between sexes increased in the two most recent decades (Table S1).

The proportion of patients reported with a primary diagnosis of SLL increased gradually from 0.2% (2 cases) in 1973–1982 to 11% (306 cases) in 2003–2012 (Table S2).

### Incidence

Number of patients diagnosed with CLL increased from 351 patients in 1953–1962 to 2636 patients in 2003–2012 (Table 1, Figure 1). There was a 68% increase in incidence between 1993–2002 (1557 patients) and 2003–2012 (2636 patients). We analyzed age‐standardized incidence rates year by year, and we found a sudden increase in incidence rates from 1.7/100.000 person‐years in 2001 to 2.6 /100.000 person‐years in 2002. (Table S3).

We found the change in age‐standardized incidence trends statistically significant when using Joinpoint Regression Program. From 2000 to 2003, the annual incidence rates rose from 1.6/100.000 to 2.8/100.000 person‐years (annual percentage change 24.9; CI: 4.3–49.5; *P* < 0.01) (Fig. S1).

### Basis for diagnosis

The basis for diagnosis has changed dramatically during the study period. Between 1953 and 1992, most cases were diagnosed by histology and a substantial proportion (14–19%) was diagnosed at autopsy. At present, immunophenotyping by flow cytometry has become the most important diagnostic method (Table S4).

### Survival

Of the 7154 assessable patients, 5444 (76%) have died by the end of the study on June 15, 2015. Median observed survival for the whole series was 5.7 years (range: 0.7–43 years) from diagnosis, and 25% of patients survived 10 years or longer. Kaplan–Meier survival curves according to calendar periods are depicted in Fig. S2. Median survival has increased after 1973–1982 (Table 2). Men had a significantly lower median survival than women until 2002, but during the 2003–2012 period, we found no difference in median survival between men and women (Table 2).

**Table 2:** Median observed survival in years by age group, sex, and period of diagnosis

		Age group				Men	Women	*P*‐value^a^
	Overall	0–59	60–69	70–79	80+			
1953–1962	3.0	6.3	3.5	1.9	0.9	2.9	3.2	0.04565
1963–1972	2.8	4.8	3.5	2.0	1.4	2.7	3.3	<0.01
1973–1982	3.3	6.5	4.9	2.7	1.4	3.0	3.9	<0.01
1983–1992	4.2	9.8	7.3	3.9	1.6	3.9	4.8	<0.01
1993–2002	5.3	21.3	9.3	5.3	2.3	4.9	6.1	<0.01
2003–2012	8.5	NA	11.4	7.3	3.2	8.3	8.8	

a Indicates *P*‐value for log‐rank test for equality of survivor functions in men and women. NA, not applicable.

**Figure 2:**
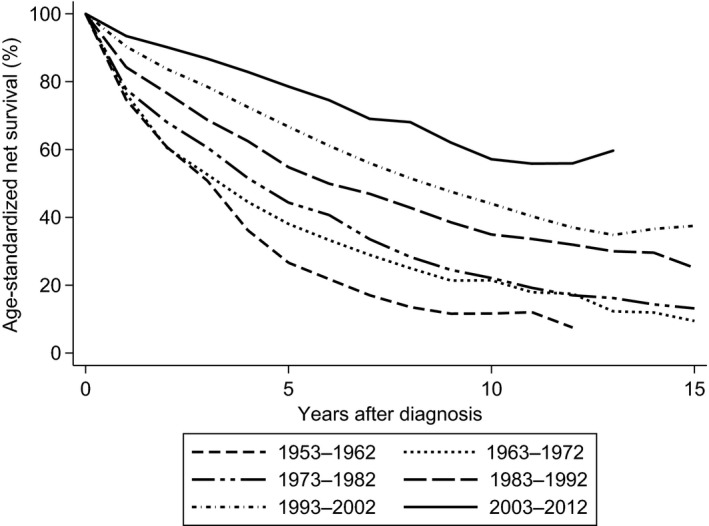
Age‐standardized net survival by periods of diagnosis.

**Figure 3:**
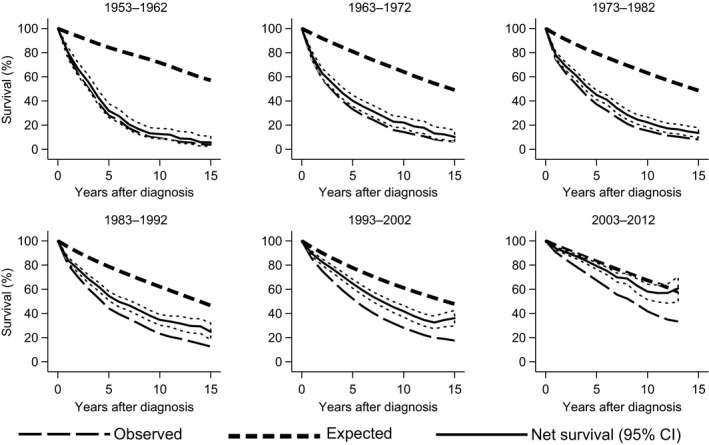
Observed‐ and net survival in patients with CLL and expected survival for a general population matched by age, sex, and calendar year at diagnosis. CLL, chronic lymphocytic leukemia.

Five‐ and 10‐year age‐standardized net survival increased throughout the period (Table 3 and Fig. 2). The 10‐year net survival rates were substantially lower than 5‐year net survival rates. Increase in 10‐year age‐standardized net survival has been persistent since 1983–1992. A marked gap in 5‐, 10‐, and 15‐year survival between the patients and the general population observed in the first three decades gradually narrowed in the most recent periods (Fig. 3).

**Table 3:** Age‐standardized 5‐, 10‐, and 15‐year net survival by periods of diagnosis

	5‐year net survival			10‐year net survival			15‐year net survival		
	%	95% CI	a	%	95% CI	a	%	95% CI	a
1953–1962	27	(21–33)		12	(6–19)		NA	NA	
1963–1972	38	(33–43)	11	21	(16–28)	9	10	(5–15)	
1973–1982	44	(41–48)	6	22	(18–27)	1	13	(10–17)	3
1983–1992	55	(51–58)	11	35	(31–39)	13	25	(19–32)	12
1993–2002	67	(63–70)	12	44	(40–48)	9	38	(31–44)	13
2003–2012	79	(76–81)	12	57	(51–63)	13	NA	NA	

a Indicates increase from the previous period in percentage points (rounded). NA, not applicable.

Five‐, 10‐, and 15‐year age‐standardized net survival of men were lower in all age groups and study periods with the exception of equal 5‐year survival of men and women (91%) in patients younger than 60 years in the most recent decade. (Tables S5 and S6). The gap between 5‐ and 10‐year net survival of men and women tends to decrease in the most recent decade in patients younger than 60 years while it persists in older patients (Figs S3 and S4).

Comparison of relative survival and net survival is provided in Data S6 on page 8 and 9.

## Discussion

Our study revealed a fourfold increase in the age‐standardized incidence of CLL in Norway during 60 years (1953–2012) with an upsurge in incidence in younger patients from 2002.

The increasing incidence of CLL in Norway is consistent with similar changes described in USA, Spain, and Denmark 3, 5, 21. The steepness in the incidence curve in 2002 is probably not consistent with a change in the natural history of the disease. An important health care reform was implemented in 2002. Every Norwegian was assigned a statutory regular general practitioner. Accessibility to routine health controls improved. Automated blood cell counters became routinely available throughout Norway in the late 1990s. This may have contributed to the observed increase in the incidence of CLL. In 1998, the Norwegian Medical Association published an article on immunophenotyping in CLL in its widely distributed journal 22. Improved accessibility to the crucial diagnostic methods for CLL may explain the observed increase in incidence, particularly in younger age groups in the most recent decade. As shown in a population‐based Canadian cohort, identifying patients diagnosed solely by flow cytometry increased the age‐adjusted incidence substantially 6. The Cancer Registry of Norway has received data files based on Patient Administrative Data Systems used in all Norwegian hospitals since 2002. These files contain information on patients who have been admitted to hospitals since 1998, both inpatients and outpatients. The registry sends reminders to the clinicians when finding unreported cases in these data files. Better registration quality may contribute to the incidence increase from the late 1990s.

Flow cytometry is performed in a few centralized laboratories in Norway, including both immunology and pathology laboratories. Only pathology laboratories report directly to the Cancer Registry of Norway. Patients diagnosed by flow cytometry performed at an immunology laboratory on request of a general practitioner or a specialist in internal medicine in private practice may not be recognized by the Cancer Registry of Norway, but the number of patients will be very few. The registration in the Norwegian Cancer Registry has been considered to be close to completion 13. The completeness of the population‐based cohort is the primary strength of this study.

We confirm that CLL is shortening life expectancy. However, survival has increased, and the majority of patients diagnosed after 1993 survived more than 5 years. The cumulative age‐standardized 5‐ and 10‐year net survival in 2003–2012 was estimated to 79% and 57%, respectively. The 10‐year survival has improved since 1983, but is still considerably shorter than the 5‐year survival. Men tended to have a shorter survival than women until 2002, and in the last decade, men younger than 60 years enjoyed a longer 5‐ and 10‐year survival than women. The follow‐up in the last decade was short. Accordingly, our findings should be interpreted with caution. A new study with longer follow‐up is needed to confirm this interesting trend.

Because of lack of data on stage at diagnosis, symptoms, and treatment, we cannot closely link increase in incidence to incidental diagnoses of CLL in asymptomatic patients. The registry does not make demands on the diagnosis accuracy as it is the physicians' responsibility to follow the guidelines on diagnostic criteria for CLL. The diagnostic criteria for CLL from 1996 were used in the national guidelines valid until 2010. The guidelines were updated in 2010 according to the *iwCLL* 2008 guidelines. The iwCLL 2008 guidelines demand ≥5 × 10^9^ /L monoclonal lymphocytes with a CLL phenotype in peripheral blood. Before 2008, diagnosis of CLL relied on 1996 National Cancer Institute Working Group criteria which required an absolute lymphocyte count of 5.0 × 10^9^/L or higher 1, 23. Interestingly, we have not observed a decrease in incidence as a result of the change in guidelines in 2008 as described in Italy and USA 24, 25. Cases with monoclonal B‐cell lymphocytosis can be misdiagnosed as CLL and referred to the registry as CLL, and thus, contribute to the lack of incidence decline.

Improved survival of Norwegian patients with CLL is consistent with observations by Brenner et al. and Abrisqueta et al. 3, 26 In a Swedish cancer registry study, the 5‐year relative survival in younger patients was not improved from the 1980s to the end of the study in 2003 4. In contrast, we show gradually improved 5‐year net survival in all age groups, and, in particular, survival was improved among young men diagnosed with CLL during 2003–2012.

The national guidelines on diagnosis and treatment of CLL in Norway are based on international recommendations. Fludarabine and especially its combination with cyclophosphamide and more recently rituximab proved to prolong the overall survival in patients with symptomatic CLL and introduced the era of chemoimmunotherapy 9, 27. Because our study lacks data on treatment, we cannot link the survival prolongation to specific therapies. Aggregate‐level information on fludarabine sales in Norway from 1999 to 2013, obtained from the Norwegian Institute of Public Health (Fig. S6), show doubling of sales from 86 g in 2003 to 163 g in 2004.

A substantial increase in the annual fludarabine sales from 2003 is in line with the change in treatment recommendations in national guidelines and implies that the chemotherapy backbone of CLL therapy was intensified beyond 2002. Abrisqueta et al. reported improved survival largely due to decrease in CLL‐attributable mortality in younger patients with advanced disease in need of treatment 26. No improvement in survival was observed in patients with asymptomatic disease in this hospital cohort.

Cancer patients die from all other possible causes in addition to cancer. Reports on cause of death may be unavailable and are unreliable. Cumulative net cancer survival is an assumption of survival in a hypothetical situation where it is not possible to die from other causes than cancer. Noncancer mortality is different between countries, calendar periods, and age groups. Net cancer survival allows an “unbiased” comparison of cancer mortality between different groups. Relative survival, the ratio of the observed all‐cause survival to the expected survival has traditionally been used to estimate survival in population‐based cancer survival studies 28.

The expected mortality rates are obtained from the national survival statistics, life tables stratified by age, sex, and calendar year. The differences in age distribution between populations are taken into account by the age‐standardization, where an estimate of relative survival is calculated separately for age groups (e.g., 5‐year groups) and a weighted average is calculated using an international standard population (e.g., International cancer survival standard, ICSS). Thus, the relative survival methods are modeled estimates and dependent on the national general population mortality. Pohar‐Perme et al. developed a new, straightforward net survival estimator, which is weighting individual observation with their population survival. The Pohar‐Perme estimate eliminates the influence of other causes of death and has been proposed to be the most suitable survival estimate in population‐based cancer survival studies. We calculated cancer survival estimates by the method developed by Pohar‐Perme et al.^19^. The cumulative 5‐ and 10‐year net cancer survival by Pohar‐Perme et al. derives the proportion of cancer patients that survive up to 5 and 10 years, eliminating other causes of death than cancer. We were interested to compare the survival trends between Norway and other countries. However, the CLL survival in comparable countries has mostly been estimated by the “relative survival” methods 3, 4, 5. We provide a comparison of the traditional relative survival estimation method by Ederer (Ederer II method) and the cumulative net survival method described by Pohar‐Perme et al.^19^, ^29^. The Pohar‐Perme survival estimator tended to generate lower survival rates than the “relative survival” estimator. Though, the differences were small and possibly practically irrelevant, the confidence intervals for the Pohar‐Perme method were wider, especially at 10 and 15 years. Similar differences between results derived by those two methods have been reported in simulation settings 18.

Our study shows a steady increase in incidence of CLL and improved long‐term survival in a population‐based retrospective study from Norway. We provide some likely explanations, but more studies are needed to fully unravel the underlying mechanisms of these trends.

## Conflicts of Interest

The authors declare no competing interests.

## Supporting information


**Data S1**. Methods and Results.
**Table S1.** Median age at diagnosis (years, rounded) and age difference between men and women by calendar period.
**Table S2.** Number of patients presenting with small lymphocytic lymphoma (SLL) by calendar period.
**Data S3.** Incidence.
**Table S3.** Annual age‐standardized (world standard population) incidence of CLL in Norway.
**Figure S1.** Trend in the incidence of CLL by Joinpoint regression analysis.
**Data S4.** Basis for diagnosis.
**Table S4.** Basis for diagnosis by calendar period.
**Data S5.** Age‐standardized net survival by Pohar‐Perme et al. method.
**Table S5.** Age‐standardized net survival in women and men by period of diagnosis.
**Table S6.** Five‐, 10‐, and 15‐year age‐standardized net survival by sex, age group, and period of diagnosis.
**Figure S2**. Kaplan–Meier survival estimates by period of diagnosis.
**Figure S3.** Age‐standardized net survival of women and men diagnosed at an age younger than 60 years, by period of diagnosis.
**Figure S4.** Age‐standardized net survival of women and men diagnosed at age 60 years and older by period of diagnosis.
**Data S6.** Comparison of relative and net survivals.
**Table S7.** Five‐, 10‐, and 15‐year cumulative relative survival (Ederer II method) and cumulative net survival ( Pohar‐Perme et al. method) of patients with CLL by period of diagnosis.
**Figure S5.** Cumulative relative survival (Ederer II method) and cumulative net survival (Pohar‐Perme et al. method) by period of diagnosis.
**Figure S6.** Aggregate fludarabine sales in Norway.
