# Supplementary material for: National trends in incidence and survival of chronic lymphocytic leukemia in Norway for 1953–2012: a systematic analysis of population‐based data
Source: Cancer Med. 2016 Nov 4;5(12):3588–95. doi: 10.1002/cam4.849 (PMC5224846; doi:10.1002/cam4.849)

**Methods**

**Patient cohort**

The extraction of data from the Cancer Registry of Norway included following codes:

From period 1953-1992: MoTNaC-codes 9827 and 1114.

From period 1993-2012: 2^nd^ edition of International Classification of Diseases of Oncology codes 131039, 131139, 131239, 131339, 131439, 141139, 220339, 967039 and 982339.

**Results**

**The study population**

| **Table A1. Median age at diagnosis (years, rounded) and age difference between men and women by calendar period.** | | | | | | | |
| --- | --- | --- | --- | --- | --- | --- | --- |
|  | | | | | | | |
| **Periods** | **Both sexes** |  | **Women** | **Men** | **Difference** | **P-value** |  |
| 1953-1962 | 68 |  | 69 | 68 | 1.17 | 0.2894 |  |
| 1963-1972 | 69 |  | 71 | 68 | 2.54 | <0.01 |  |
| 1973-1982 | 72 |  | 73 | 71 | 2.41 | <0.01 |  |
| 1983-1992 | 73 |  | 75 | 72 | 2.38 | <0.01 |  |
| 1993-2002 | 75 |  | 78 | 74 | 3.92 | <0.01 |  |
| 2003-2012 | 72 |  | 74 | 70 | 4.79 | <0.01 |  |
| **1953-2012** | 72 |  | 74 | 71 | 3.42 | <0.01 |  |

**Table A2. Number of patients presenting with small lymphocytic lymphoma (SLL) by calendar period.**

| **Calendar period** | **Cases with SLL** | **All CLL cases** |
| --- | --- | --- |
| 1973-1982 | 2 | 1139 |
| 1983-1992 | 83 | 1339 |
| 1993-2002 | 133 | 1557 |
| 2003-2012 | 306 | 2636 |

**Incidence**

**Table A3. Annual age-standardized (world standard** population) incidence of CLL in Norway.

| **Year** | **Number of patients** | **Age-standardized incidence (rounded)** |
| --- | --- | --- |
| 1953 | 28 | 0.6 |
| 1954 | 21 | 0.5 |
| 1955 | 30 | 0.7 |
| 1956 | 42 | 0.9 |
| 1957 | 30 | 0.6 |
| 1958 | 37 | 0.7 |
| 1959 | 42 | 0.8 |
| 1960 | 30 | 0.6 |
| 1961 | 42 | 0.8 |
| 1962 | 49 | 0.9 |
| 1963 | 36 | 0.7 |
| 1964 | 57 | 1.0 |
| 1965 | 56 | 0.9 |
| 1966 | 70 | 1.1 |
| 1967 | 66 | 1.1 |
| 1968 | 69 | 1.1 |
| 1969 | 59 | 0.9 |
| 1970 | 73 | 1.1 |
| 1971 | 78 | 1.1 |
| 1972 | 78 | 1.2 |
| 1973 | 83 | 1.3 |
| 1974 | 120 | 1.7 |
| 1975 | 116 | 1.6 |
| 1976 | 107 | 1.5 |
| 1977 | 110 | 1.5 |
| 1978 | 122 | 1.6 |
| 1979 | 115 | 1.4 |
| 1980 | 124 | 1.7 |
| 1981 | 99 | 1.3 |
| 1982 | 143 | 1.9 |
| 1983 | 129 | 1.6 |
| 1984 | 125 | 1.6 |
| 1985 | 125 | 1.6 |
| 1986 | 125 | 1.6 |
| 1987 | 116 | 1.3 |
| 1988 | 118 | 1.5 |
| 1989 | 161 | 1.9 |
| 1990 | 125 | 1.6 |
| 1991 | 160 | 1.9 |
| 1992 | 155 | 1.8 |
| 1993 | 159 | 1.8 |
| 1994 | 143 | 1.6 |
| 1995 | 166 | 1.7 |
| 1996 | 144 | 1.6 |
| 1997 | 139 | 1.5 |
| 1998 | 136 | 1.5 |
| 1999 | 144 | 1.6 |
| 2000 | 136 | 1.6 |
| 2001 | 157 | 1.7 |
| 2002 | 233 | 2.6 |
| 2003 | 241 | 2.8 |
| 2004 | 248 | 2.8 |
| 2005 | 258 | 2.8 |
| 2006 | 212 | 2.4 |
| 2007 | 245 | 2.7 |
| 2008 | 234 | 2.6 |
| 2009 | 283 | 3.0 |
| 2010 | 295 | 3.1 |
| 2011 | 322 | 3.4 |
| 2012 | 298 | 3.1 |

**Figure A1. Trend in the incidence of CLL by Joinpoint regression analysis.**

**
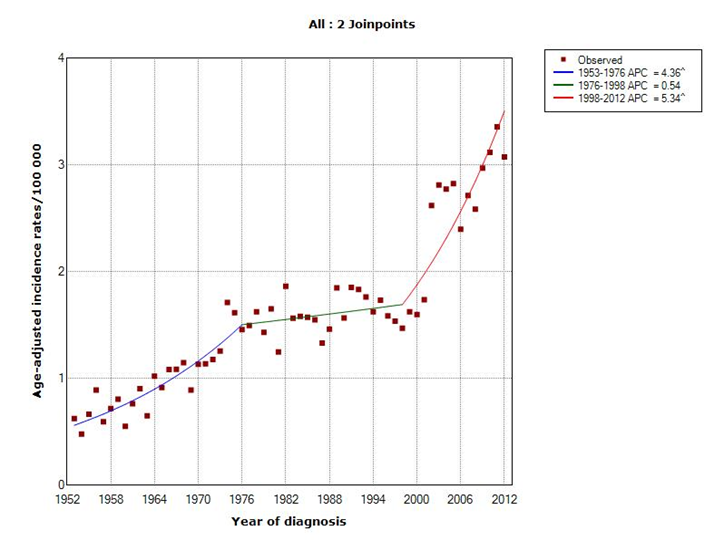
**

^ The annual percentage change (APC) is significantly different from zero at alpha=0·05.

**Basis for diagnosis**

**Table A4.** Basis for diagnosis by calendar period.

|  | **Immun.(%)** | **Cytology (%)** | **Histology (%)** | **Autopsy (%)** | **Other (%)** |
| --- | --- | --- | --- | --- | --- |
| 1953-1962 | 0 | 13 (4) | 268 (76) | 67 (19) | 3 (<1) |
| 1963-1972 | 1 (<1) | 43 (7) | 486 (76) | 97 (15) | 15 (2) |
| 1973-1982 | 1 (<1) | 27 (2) | 891 (78) | 160 (14) | 60 (5) |
| 1983-1992 | 11 (<1) | 105 (8) | 999 (75) | 33 (3) | 191 (14) |
| 1993-2002 | 542 (35) | 613 (39) | 204 (13) | 9 (<1) | 189 (12) |
| 2003-2012 | 1491 (57) | 445 (17) | 60 (2) | 1 (<1) | 639 (24) |

“Immun.” indicates immunophenotyping .”Other” includes clinical and radiological diagnosis, genetics as basis for diagnosis, death certificate based diagnosis and unknown basis for diagnosis.

**Age standardized net survival by Pohar-Perme et al. method**

**Table A5. Age-standardized net survival in women and men by period of diagnosis.**

|  | **Overall** | | **Men** | | **Women** | |
| --- | --- | --- | --- | --- | --- | --- |
|  | **Survival (%)** | **95% CI** | **Survival (%)** | **95% CI** | **Survival (%)** | **95% CI (%)** |
| **5-year survival** |  |  |  |  |  |  |
| 1953-1962 | 27 | (21-33) | NA |  | 28 | (18-38) |
| 1963-1972 | 38 | (33-43) | 34 | (28-40) | 44 | (36-52) |
| 1973-1982 | 44 | (41-48) | 40 | (35-44) | 51 | (46-57) |
| 1983-1992 | 55 | (51-58) | 50 | (45-55) | 61 | (56-66) |
| 1993-2002 | 67 | (63-70) | 62 | (57-66) | 74 | (69-79) |
| 2003-2012 | 79 | (76-81) | 76 | (72-80) | 82 | (78-85) |
| **10-year survival** |  |  |  |  |  |  |
| 1953-1962 | 12 | (6-19) | NA |  | 16 | (5-30) |
| 1963-1972 | 21 | (16-28) | 14 | (9-19) | 33 | (22-45) |
| 1973-1982 | 22 | (18-27) | 18 | (13-24) | 28 | (22-34) |
| 1983-1992 | 35 | (31-39) | 31 | (26-37) | 41 | (35-47) |
| 1993-2002 | 44 | (40-48) | 40 | (34-47) | 51 | (45-57) |
| 2003-2012 | 57 | (51-63) | 55 | (45-63) | 62 | (54-69) |
| **15-year survival** |  |  |  |  |  |  |
| 1953-1962 | NA |  | NA |  | NA |  |
| 1963-1972 | 10 | (5-15) | NA |  | 15 | (7-27) |
| 1973-1982 | 13 | (10-17) | NA |  | 19 | (14-26) |
| 1983-1992 | 25 | (19-32) | 24 | (14-35) | 28 | (21-35) |
| 1993-2002 | 38 | (31-44) | 39 | (28-50) | 39 | (31-46) |
| 2003-2012 | NA | NA | NA |  | NA |  |

“NA”(not applicable)

**Table A6. Five-, 10- and 15-year age-standardized net survival by sex, age group and period of diagnosis.**

|  | **0-59** |  |  | **60-69** |  |  | **70-79** |  |  | **80+** |  |
| --- | --- | --- | --- | --- | --- | --- | --- | --- | --- | --- | --- |
|  | **%(95% CI)** |  |  | **%(95% CI)** |  |  | **%(95% CI)** |  |  | **%(95% CI)** |  |
| **5-year net survival** | **Women** | **Men** |  | **Women** | **Men** |  | **Women** | **Men** |  | **Women** | **Men** |
| 1953-1962 | 61 (50-71) | NA |  | 26 (17-34) | 36 (28-45) |  | 22 (9-38) | 11 (5-19) |  | 15 (3-36) | 21 (5-44) |
| 1963-1972 | 57 (46-67) | 48 (42-54) |  | 44 (35-52) | 42 (36-49) |  | 41 (29-52) | 31 (22-41) |  | 43 (26-59) | 12 (5-22) |
| 1973-1982 | 74 (66-80) | 62 (57-67) |  | 66 (59-73) | 48 (42-53) |  | 48 (39-56) | 33 (26-41) |  | 21 (14-30) | 21 (14-29) |
| 1983-1992 | 77 (68-83) | 70 (64-74) |  | 75 (68-80) | 63 (57-69) |  | 57 (49-64) | 47 (40-54) |  | 38 (30-47) | 24 (17-33) |
| 1993-2002 | 92 (86-95) | 88 (82-90) |  | 89 (82-93) | 70 (64-75) |  | 76 (68-81) | 58 (51-64) |  | 49 (42-57) | 39 (31-47) |
| 2003-2012 | 91 (88-94) | 91 (88-93) |  | 95 (91-97) | 87 (83-90) |  | 85 (79-90) | 75 (68-80) |  | 61 (53-68) | 50 (40-58) |
| **10-year net survival** |  |  |  |  |  |  |  |  |  |  |  |
| 1953-1962 | 31 (21-41) | NA |  | 12 (6-21) | 10 (5-16) |  | NA | NA |  | 38 (3-76) | NA |
| 1963-1972 | 45 (34-56) | 26 (21-32) |  | 30 (21-39) | 13 (8-18) |  | 33 (19-49) | 15 (7-25) |  | 33 (10-59) | NA |
| 1973-1982 | 43 (35-50) | 32 (27-38) |  | 35 (28-42) | 22 (16-27) |  | 30 (21-40) | 16 (9-25) |  | 5 (1-13) | 6 (1-21) |
| 1983-1992 | 68 (59-76) | 47 (41-53) |  | 55 (48-62) | 39 (33-46) |  | 36 (27-45) | 25 (17-33) |  | 14 (6-24) | 20 (9-35) |
| 1993-2002 | 77 (69-83) | 65 (59-71) |  | 69 (60-76) | 48 (42-54) |  | 44 (35-51) | 32 (24-40) |  | 24 (15-33) | 26 (14-40) |
| 2003-2012 | 84 (78-89) | 83 (79-87) |  | 78 (67-85) | 63 (56-70) |  | 56 (44-66) | 44 (33-54) |  | 36 (21-50) | 44 (21-65) |
| **15-year net survival** |  |  |  |  |  |  |  |  |  |  |  |
| 1953-1962 | 16 (9-24) | NA |  | NA | 12 (5-21) |  | NA | NA |  |  |  |
| 1963-1972 | 28 (19-39) | 20 (15-26) |  | 18 (10-27) | 7 (3-13) |  | 16 (3-39) | NA |  |  |  |
| 1973-1982 | 33 (26-40) | 19 (14-23) |  | 28 (21-36) | 15 (9-21) |  | 18 (8-31) | NA |  |  |  |
| 1983-1992 | 52 (42-61) | 30 (25-36) |  | 35 (28-42) | 29 (22-36) |  | 26 (15-40) | 29 (16-43) |  | 8 (1-23) | 4 (1-27) |
| 1993-2002 | 71 (62-78) | 58 (51-64) |  | 56 (45-64) | 36 (29-43) |  | 29 (20-39) | 23 (13-36) |  | 13 (4-30) | 51 (16-78) |
| 2003-2012 | NA | NA |  | NA | NA |  | NA | NA |  |  |  |
| “NA” (not applicable) | | | | | | | | | | | |

**Figure A2. Kaplan-Meier survival estimates by period of diagnosis.**

**
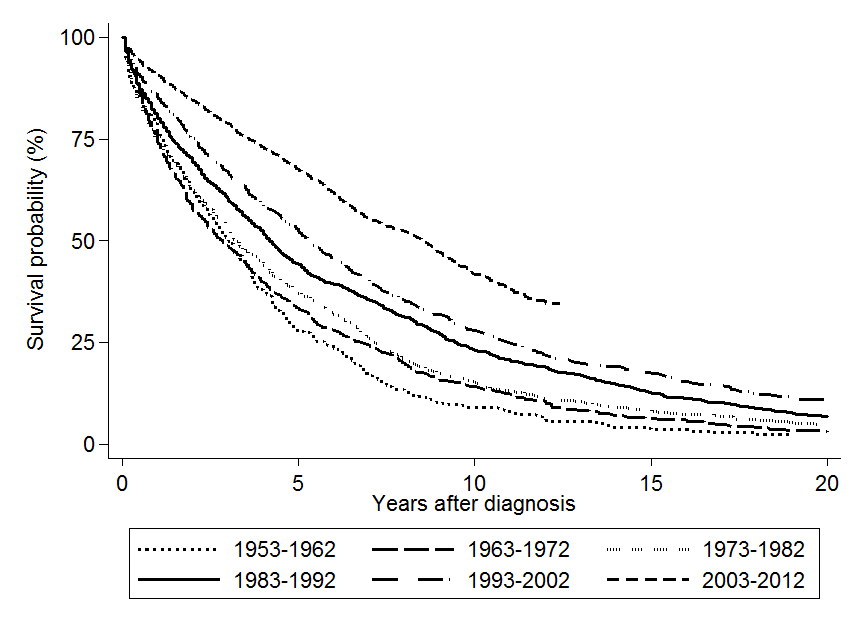
**

**Figure A3. Age-standardized net survival of women and men diagnosed at an age younger than 60 years, by period of diagnosis.**

**
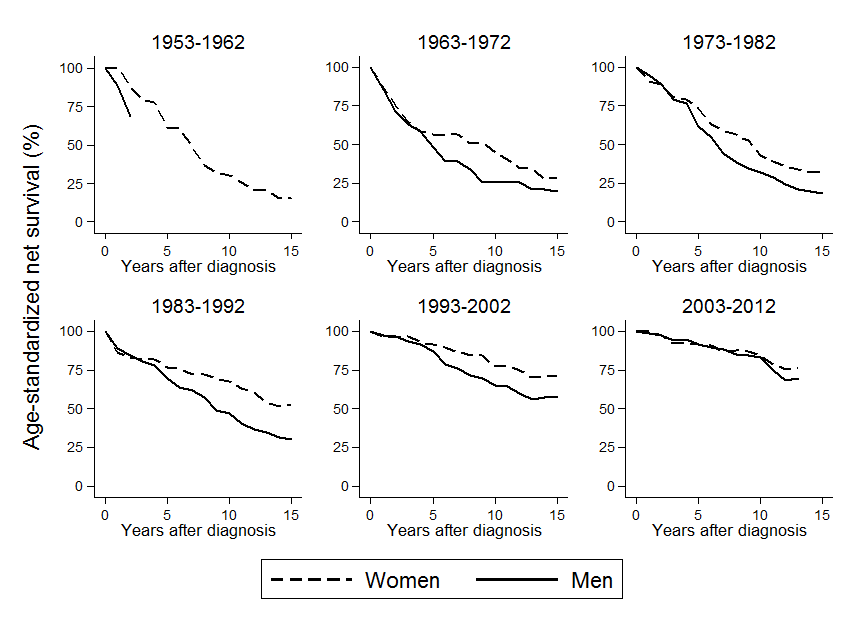
**

**Figure A4. Age-standardized net survival of women and men diagnosed at age 60 years and older by period of diagnosis.**

**
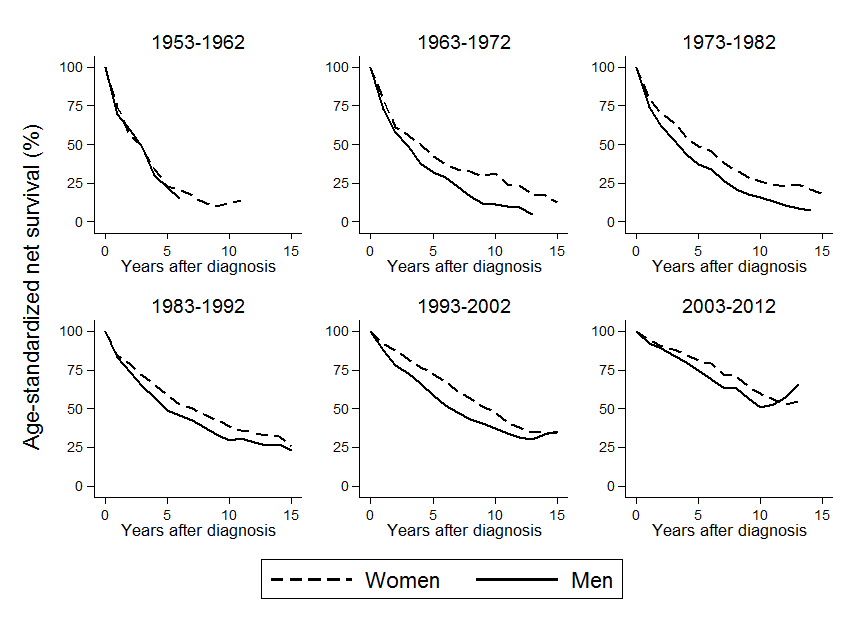
**

**Comparison of relative and net survivals**

Estimates and curves of relative survival derived by the Ederer II method and net survival derived by the Pohar-Perme et al. method are depicted in Table A7 and Figure A5.

Estimates derived by the Pohar-Perme method were 0.12 to 4.03 percentage points lower than by Ederer II method. The differences were largest in the last two decades for 10-year survival. The Pohar-Perme method derived higher standard error.

**Table A7. Five-, 10- and 15-year cumulative relative survival (Ederer II method) and cumulative net survival ( Pohar-Perme et al. method) of patients with CLL by period of diagnosis.**

|  | **Relative survival (%)** | **Net survival (%)** | ***** | **SE E-II** | **SE PP** |
| --- | --- | --- | --- | --- | --- |
| **5-year survival** |  |  |  |  |  |
| 1953-1962 | 33.25 | 31.91 | 1.34 | 2.89 | 2.93 |
| 1963-1972 | 41.02 | 40.16 | 0.86 | 2.35 | 2.40 |
| 1973-1982 | 46.95 | 44.99 | 1.96 | 1.88 | 1.97 |
| 1983-1992 | 56.10 | 54.48 | 1.62 | 1.82 | 1.94 |
| 1993-2002 | 67.19 | 64.70 | 2.49 | 1.71 | 1.88 |
| 2003-2012 | 81.12 | 79.29 | 1.83 | 1.18 | 1.36 |
| **10-year survival** |  |  |  |  |  |
| 1953-1962 | 12.76 | 12.60 | 0.16 | 2.18 | 2.19 |
| 1963-1972 | 22.31 | 22.17 | 0.14 | 2.20 | 2.38 |
| 1973-1982 | 24.35 | 22.32 | 2.03 | 1.77 | 2.14 |
| 1983-1992 | 37.34 | 34.66 | 2.68 | 1.96 | 2.26 |
| 1993-2002 | 45.82 | 41.79 | 4.03 | 1.95 | 2.43 |
| 2003-2012 | 61.94 | 57.05 | 3.44 | 2.15 | 3.26 |
| **15-year survival** |  |  |  |  |  |
| 1953-1962 | 6.72 | 5.76 | 0.96 | 1.83 | 2.16 |
| 1963-1972 | 12.92 | 10.23 | 2.69 | 2.00 | 2.54 |
| 1973-1982 | 16.82 | 13.55 | 3.27 | 1.74 | 2.08 |
| 1983-1992 | 27.08 | 24.75 | 2.33 | 2.06 | 3.57 |
| 1993-2002 | 36.62 | 36.05 | 0.12 | 2.18 | 3.29 |
| 2003-2012 | NA | NA |  | NA | NA |

*indicates difference in percentage points, “SE E-II” (standard error of Ederer-II estimates), “SE PP” (standard error of Pohar-Perme estimates), “NA”(not applicable)

**Figure A5. Cumulative relative survival (Ederer II method) and cumulative net survival (Pohar –Perme et al method) by period of diagnosis.**


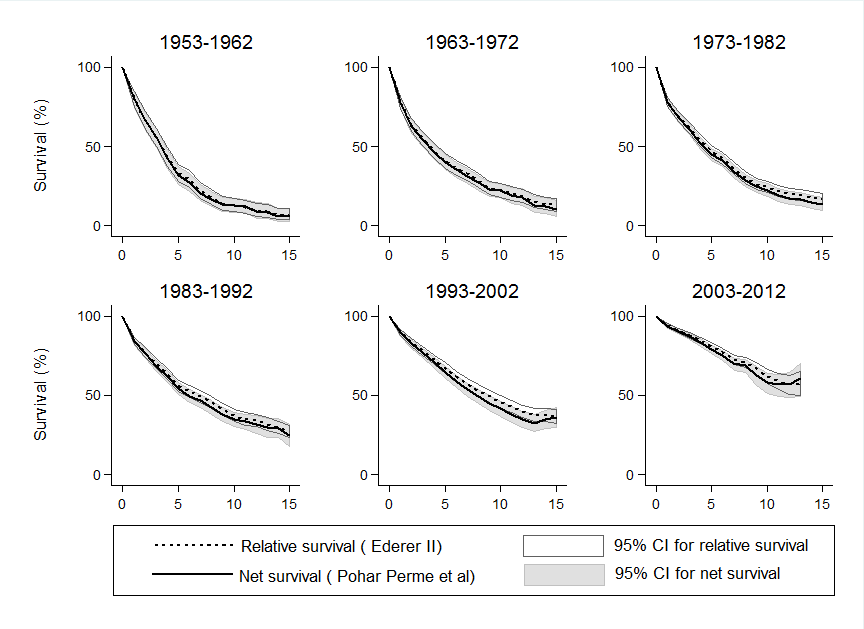


**Figure A6. Aggregate fludarabine sales in Norway.**


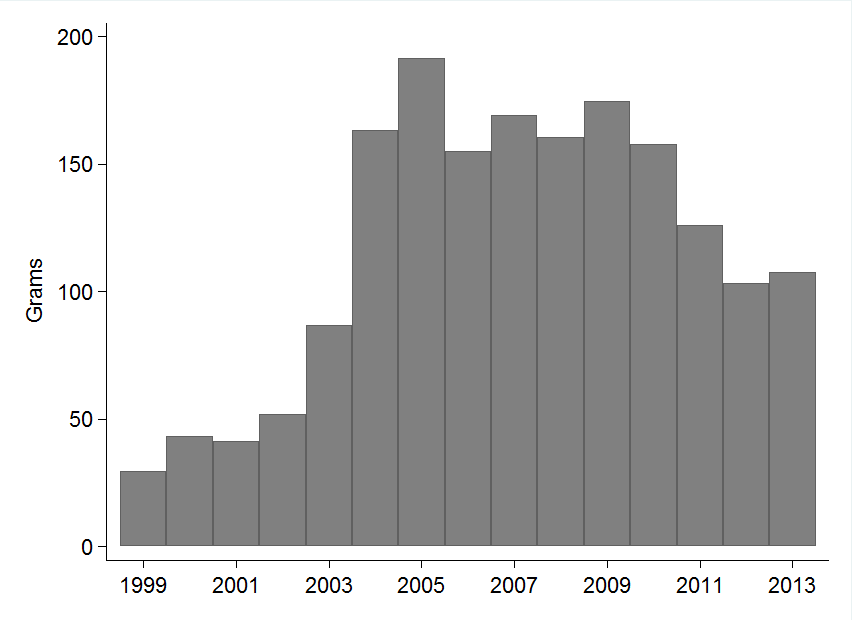

Supplement: Supplementary file 1 — Data S1. Methods and Results. Table S1. Median age at diagnosis (years, rounded) and age difference between men and women by calendar period. Table S2. Number of patients presenting with small lymphocytic lymphoma (SLL) by calendar period. Data S3. Incidence. Table S3. Annual age‐standardized (world standard population) incidence of CLL in Norway. Figure S1. Trend in the incidence of CLL by Joinpoint regression analysis. Data S4. Basis for diagnosis. Table S4. Basis for diagnosis by calendar period. Data S5. Age‐standardized net survival by Pohar‐Perme et al. method. Table S5. Age‐standardized net survival in women and men by period of diagnosis. Table S6. Five‐, 10‐, and 15‐year age‐standardized net survival by sex, age group, and period of diagnosis. Figure S2. Kaplan–Meier survival estimates by period of diagnosis. Figure S3. Age‐standardized net survival of women and men diagnosed at an age younger than 60 years, by period of diagnosis. Figure S4. Age‐standardized net survival of women and men diagnosed at age 60 years and older by period of diagnosis. Data S6. Comparison of relative and net survivals. Table S7. Five‐, 10‐, and 15‐year cumulative relative survival (Ederer II method) and cumulative net survival ( Pohar‐Perme et al. method) of patients with CLL by period of diagnosis. Figure S5. Cumulative relative survival (Ederer II method) and cumulative net survival (Pohar‐Perme et al. method) by period of diagnosis. Figure S6. Aggregate fludarabine sales in Norway. [file CAM4-5-3588-s001.doc]
